# Unmet needs: therapeutic standards of care

**DOI:** 10.1186/ar4221

**Published:** 2013-07-11

**Authors:** Bevra Hahn

**Affiliations:** 1 Professor Emerita of Medicine, David Geffen School of Medicine at UCLA, Division of Rheumatology, Department of Medicine, Los Angeles, California, USA

## 

Many unmet needs exist for practitioners caring for patients with systemic lupus erythematosus (SLE). The top need is for a blockbuster drug with glucocorticoid efficacy but fewer side effects. They also need better markers to identify responders without waiting 3 to 6 months to see clinical improvement. Current therapeutic goals are to induce and maintain responses while minimizing side effects. Therapy selection varies depending on disease severity and response to previous therapies. Therapies for SLE target different areas in the immunologic process, primarily T-cell and B-cell lymphocytes for mycophenolate mofetil (MMF), azathioprine, and anti-B-lymphocyte stimulator, and a wider array of cells for glucocorticoids. The American College of Rheumatology recently published treatment guidelines for the use of adjunctive therapies in patients with lupus nephritis that recommend hydroxychloroquine as background therapy in all of these patients, and it established target levels for blood pressure, and low-density lipoprotein to reduce complications. Data were inadequate to make recommendations regarding glucocorticoid doses for induction of improvement (thus 0.5 to 1 mg/kg/day is the recommendation) or for tapering or discontinuing prednisone. Renal biopsy is recommended for all patients with active lupus nephritis, previously untreated, to provide data for classification of glomerular disease and thus enable selection of appropriate therapy. Recommendations for induction therapy include either MMF or cyclophosphamide (CYC). MMF has the advantage of being equally effective in all races and being formulated for oral administration. CYC is less effective in African Americans and Latinos but is equivalent in Caucasians and Asians. Low-dose CYC is an option for Caucasians. Teratogenicity is a concern with MMF, CYC, and methotrexate, but hydroxychloroquine, glucocorticoids, and azathioprine can be used during pregnancy, if necessary. New evidence suggests that improvements in proteinuria and C3/C4 blood levels may predict response as early as 8 weeks. Clinical trials with belimumab for lupus nephritis are not yet available although it did not appear to worsen renal disease in patients without active lupus nephritis. Belimumab is approved by the US Food and Drug Administration for use in antinuclear antibodies and/or anti-DNA-positive SLE patients with active disease that persists despite standard treatments. Investigators continue to research other targeted therapies for high response rates, less toxicity, and less need for chronic glucocorticoid treatment.

Additional file 1 contains an edited transcript of the full presentation.

## Supplementary Material

Additional file 1 Click here for file

