# Supplementary material for: Unmet needs: therapeutic standards of care
Source: Arthritis Res Ther. 2013 Jul 11;15(Suppl 2):A3. doi: 10.1186/ar4221 (PMC3891353; doi:10.1186/ar4221)
Supplement: Additional file 1 [file ar4221-S1.pdf]

Presentation

Unmet needs: therapeutic standards of care

Bevra Hahn

Professor Emerita of Medicine, David Geffen School of Medicine at UCLA, Division of Rheumatology, Department of Medicine, Los Angeles, California, USA

Abstract

Many unmet needs exist for practitioners caring for patients with systemic lupus erythematosus (SLE). The top need is for a blockbuster drug with glucocorticoid efficacy but fewer side effects. They also need better markers to identify responders without waiting 3 to 6 months to see clinical improvement. Current therapeutic goals are to induce and maintain responses while minimizing side effects. Therapy selection varies depending on disease severity and response to previous therapies. Therapies for SLE target different areas in the immunologic process, primarily T-cell and B-cell lymphocytes for mycophenolate mofetil (MMF), azathioprine, and anti-B-lymphocyte stimulator, and a wider array of cells for glucocorticoids. The American College of Rheumatology recently published treatment guidelines for the use of adjunctive therapies in patients with lupus nephritis that recommend hydroxychloroquine as background therapy in all of these patients, and it established target levels for blood pressure, and low-density lipoprotein to reduce complications. Data were inadequate to make recommendations regarding glucocorticoid doses for induction of improvement (thus 0.5 to 1 mg/kg/day is the recommendation) or for tapering or discontinuing prednisone. Renal biopsy is recommended for all patients with active lupus nephritis, previously untreated, to provide data for classification of glomerular disease and thus enable selection of appropriate therapy. Recommendations for induction therapy include either MMF or cyclophosphamide (CYC). MMF has the advantage of being equally effective in all races and being formulated for oral administration. CYC is less effective in African Americans and Latinos but is equivalent in Caucasians and Asians. Low-dose CYC is an option for Caucasians. Teratogenicity is a concern with MMF, CYC, and methotrexate, but hydroxychloroquine, glucocorticoids, and azathioprine can be used during pregnancy, if necessary. New evidence suggests that improvements in proteinuria and C3/C4 blood levels may predict response as early as 8 weeks. Clinical trials with belimumab for lupus nephritis are not yet available although it did not appear to worsen renal disease in patients without active lupus nephritis. Belimumab is approved by the US Food and Drug Administration for use in antinuclear antibodies and/or anti-DNA-positive SLE patients with active disease that persists despite standard treatments. Investigators continue to research other targeted therapies for high response rates, less toxicity, and less need for chronic glucocorticoid treatment.

Disclosures

About this presentation
This presentation was developed from an audio transcript of Dr. Hahn's presentation at the "Treatment of SLE: Bridging the Gap from Clinical Trials to Practice" symposium held during the Annual Congress of the American College of Rheumatology on November 11, 2012.

The transcript was formatted and edited by Cleveland Clinic and BioMed Central staff for clarity and conciseness, and was then reviewed, revised, and approved by Dr. Hahn.

Sponsorship
The Cleveland Clinic Foundation Center for Continuing Education acknowledges an educational grant for support of this activity from Human Genome Sciences.

Accreditation
The Cleveland Clinic Foundation Center for Continuing Education is accredited by the Accreditation Council for Continuing Medical Education to provide continuing medical education for physicians. The Cleveland Clinic Foundation Center for Continuing Education designates these (4) enduring activities for a maximum of **1.0** *AMA PRA Category 1 Credit*™. Physicians should claim only the credit commensurate with the extent of their participation in the activity. Participants claiming CME credit from this activity may submit the credit hours to the American Osteopathic Association for Category 2 credit.

To claim CME credit, visit www.ccfcme.org/SLECMEsupplement. CME credit may be obtained upon reading all four CME-certified presentations online.

Author disclosures
In accordance with the Standards for Commercial Support issued by the Accreditation Council for Continuing Medical Education (ACCME), The Cleveland Clinic Foundation Center for Continuing Education requires resolution of all faculty conflicts of interest to ensure CME activities are free of commercial bias. Dr. Hahn has indicated she has no relationship which, in the context of her presentation(s), could be perceived as a potential conflict of interest.

All other planners, CME staff, and content reviewers, have no relevant financial relationships to disclose.

This presentation has not been subject to peer review. The statements and opinions expressed herein are those of the author, who bears full responsibility for the content of this presentation.

INTRODUCTION

Healthcare practitioners who care for patients with systemic lupus erythematosus (SLE) face a number of challenges in improving treatment outcomes. Recent guidelines from the American College of Rheumatology (ACR) provide some guidance but questions remain regarding how to incorporate them into clinical practice. Current therapies for SLE fall short of meeting the needs of practitioners in several areas.

Therapies

The number one need is for a new blockbuster drug, something with similar response rates to high-dose glucocorticoids but with substantially fewer side effects. These new drugs need to have response rates in the 90% range, rather than in the 50% range, and need to work fast; affect more pathways, be corticosteroid sparing, and, finally, the treatments need to be safer.

Adverse effects

Therapies need to be better at preventing damage associated with their use. This includes protection from infection, bone loss, hypertension, diabetes, and accelerated atherosclerosis, all substantial concerns with current therapeutic options.

Biologic and/or genetic markers

Practitioners need better markers so they can identify likely responders both before selecting therapy and shortly after starting therapy without waiting 3 to 6 months to see responses. Markers need to enable practitioners to identify flares before they occur, not when they are clinically obvious. Markers also need to identify patients who are at increased risk for experiencing adverse effects from the treatments or irreversible organ damage.

THERAPEUTIC GOALS

Goals of SLE therapies can be summarized into three areas:

- inducing improvement,
- maintaining that improvement, and
- minimizing damage from the therapies as well as from other adverse events associated with SLE.

Disease severity can be used to make treatment selections, with different drug options for each category.

Mild to moderate SLE. For these patients, the current standard therapies are hydroxychloroquine or other antimalarials. Analgesics are used for local treatment of the skin and joints. Low-dose glucocorticoids are used to improve patients' quality of life. Lastly, belimumab is being evaluated as an option for therapy.

Severe or life-threatening disease. High-dose glucocorticoids are the first-line therapy, but doses should be tapered so that the high doses are limited to short-term use. Other therapeutic options include mycophenolate mofetil (MMF), cyclophosphamide (CYC), azathioprine, and belimumab.

Poor responders to standard treatments. In these patients, therapeutic choices include rituximab, calcineurin inhibitors, and drug combinations.

Several therapeutic classes are US Food and Drug Administration approved for SLE including glucocorticoids, hydroxychloroquine, and the anti-B-lymphocyte stimulator (BLyS) belimumab. Therapies used off-label include MMF, CYC, methotrexate, azathioprine, rituximab, rapamycin and the calcineurin inhibitors cyclosporine or tacrolimus.

The following therapies target different areas in the immunologic process:

- Glucocorticoids affect both innate immunity (macrophages, monocytes, neutrophils, dendritic cells) and T-cell and B-cell lymphocytes.
- Hydroxychloroquine is thought to affect primarily antigen presentation (monocytes and/or macrophages, dendritic cells, B cells), as well as TLR-type signaling in innate immune cells and B cells.
- Anti-BLyS therapy targets B-cell maturation with subsequent decline in mature B cells and the plasmablasts/plasma cells into which they differentiate to secrete autoantibodies.
- MMF is highly specific toward preventing T-lymphocyte and B-lymphocyte proliferation but works without real direct effects on this innate immune system.
- CYC kills any rapidly dividing cells, including lymphocytes, ova, sperm, hair, and cells lining the gastrointestinal tract.
- Azathioprine also prevents cell division, particularly of lymphocytes.

Specific pathways in target cells are inhibited by MMF and anti-BLyS. In contrast, glucocorticoids target multiple pathways, an area in which more agents are needed.

AMERICAN COLLEGE OF RHEUMATOLOGY TREATMENT GUIDELINES

The ACR developed guidelines for the use of adjunctive therapies in patients with lupus nephritis that were published in June 2012 [1]. The new guidelines detail several important recommendations:

- Hydroxychloroquine is recommended as background therapy for all SLE patients with lupus nephritis unless there is a major contraindication. This opinion-level recommendation was based on evidence showing that hydroxychloroquine reduced flares in SLE patients and had less damage accrual, including renal damage.
- Angiotensin-converting enzyme inhibitors or angiotensin-receptor blockers are recommended for lupus nephritis patients with proteinuria of at least 0.5 g/24 hours, which can be measured in a 24-hour urine collection or as a protein:creatinine ratio in a single urine specimen. This recommendation was based on randomized controlled trials showing that these agents significantly reduced proteinuria and prevented or reduced end-stage renal disease.

This presentation has not been subject to peer review. The statements and opinions expressed herein are those of the author, who bears full responsibility for the content of this presentation.

- Hypertension control is defined as maintaining a target blood pressure of 130/80 mmHg or below. Randomized controlled trials have shown that blood pressure control using this target reduces end-stage renal disease in patients with various types of lupus nephritis.
- Statin therapy is recommended in patients with low-density lipoprotein levels >100 mg/dl. However, this is an opinion-level recommendation; evidence of efficacy in lupus nephritis patients is limited.
- Pregnancy counseling is recommended for all fertile women. This recommendation is obviously needed, but it is not supported by any clinical trials.

Clinical experience has shown that SLE, especially lupus nephritis, acts differently in different populations. Note that the evidence base for hydroxychloroquine included the LUMINA Study Group [2,3], which recruited a substantial number of African Americans and Latin Americans, populations not normally included in SLE studies. Although results were better for all participants in the hydroxychloroquine-treated group, they were not impressive – approximately one-half of the hydroxychloroquine group accrued new damage within 4 or 5 years of developing SLE. Thus, hydroxychloroquine does reduce damage, but it is not a blockbuster drug.

Glucocorticoid use

The ACR committee evaluated the data on glucocorticoid use in patients with lupus nephritis but found the data inadequate to make recommendations on the following questions:

- Are results better if treatment is started with an intravenous pulse followed by daily oral administration?
- Should the induction dose of daily oral glucocorticoids be 0.5 or 1.0 mg/kg/day? The 0.5 mg/kg/day dose is widely used in Europe; in the United States, 1 mg/kg/day is used.
- How rapidly can glucocorticoids be tapered without risking a flare? Although there are no randomized controlled trial data, clinical practice observations indicate that if the dose is tapered too quickly, flares often occur.
- In what proportion of patients can glucocorticoids be discontinued without flare or worsening chronic disease activity that is due to lupus and not to the steroids?

Renal biopsy and histology

The committee recommends renal biopsy (unless strongly contraindicated) for all patients with clinical evidence of active lupus nephritis, previously untreated, so that glomerular disease can be classified according to type based on current International Society of Nephrology/Renal Pathology Society Classification criteria (Table 1) [4]. The committee did not make a recommendation for treating patients with lupus nephritis of an unknown histologic class.

Table 1. ACR guidelines for immunosuppressive therapy based on ISN/RPS classification of lupus nephritis

Class I	Minimal mesangial LN
Class II	Mesangial proliferative LN
Class III	Focal LN (<50% of glomeruli)
III (A)	Active lesions
III (A/C)	Active and chronic lesions
III (C)	Chronic lesions
Class IV	Diffuse LN (≥50% glomeruli)
	Diffuse segmental (IV-S) or global (IV-G) LN
IV (A)	Active lesions
IV (A/C)	Active and chronic lesions
IV (C)	Chronic lesions
Class V	Membranous LN
Class VI	Advanced sclerosing LN (≥90% globally sclerosed glomeruli without residual activity)
Class I, II	No immunosuppression
Class III, IV, V	Immunosuppression recommended
Class VI	No immunosuppression

ACR, American College of Rheumatology; ISN/RPS, International Society of Nephrology/Renal Pathology Society; LN, lupus nephritis.

The committee recommended that treatment be largely based on the International Society of Nephrology/Renal Pathology Society classification. If it is a class I minimal mesangial or class II mesangial proliferative lupus nephritis, immunosuppressive therapy is not needed. If it is class VI advanced sclerosing lupus nephritis, in which more than 50% of the glomeruli are sclerosed, immunosuppressive therapy would have little benefit. Immunosuppressives are thus only recommended for lupus nephritis in class III and class IV (proliferative classes) and class V disease that has the nephrotic syndrome.

Induction therapy

Use of MMF dominates immunosuppressive therapy in clinical practice for patients with severe SLE, a use supported by clinical trial data. Therapy with MMF has been compared with CYC in a randomized controlled trial, the Aspreva Lupus Management Study [5]. The investigators compared MMF at 3 g/day against high-dose CYC delivered intravenously (0.5 to 1.0 g/m^2/month) to treat patients with lupus nephritis. After 6 months, overall results were similar. Of note, the group of mostly African Americans and Latin Americans (Hispanic, Mexican, South/Central America) had significantly better responses to MMF (which were similar to the other groups) than to CYC. This led the committee to adopt a recommendation to consider starting treatment with MMF in African American and Latin American patients.

A meta-analysis of studies using MMF (3 g/day) versus high-dose CYC (750 mg/m^2) found no differences in efficacy for complete, partial, or combined responses

This presentation has not been subject to peer review. The statements and opinions expressed herein are those of the author, who bears full responsibility for the content of this presentation.

[6]. Interestingly, the adverse events rates were similar for both treatments, which was surprising because MMF was originally thought to be safer. The CYC recipients did have more amenorrhea and alopecia, but there were no differences in mortality or in the rate of serious infections. Thus, what was supposed to be an advantage with MMF has not been supported in clinical trials.

In terms of MMF effects on extrarenal manifestations of SLE, a retrospective *post-hoc* analysis compared MMF (3 g/day) against high-dose intravenous CYC (0.5 to 1.0 g/m^2/month) [7]. Endpoints included musculoskeletal, cutaneous, systemic, and hematologic factors. Overall, results were similar in both groups, with approximately 90% response rates in the extrarenal domains. Although some researchers disagree on the interpretation, these data show that MMF provides a good treatment option for lupus nephritis.

Low-dose cyclophosphamide as an option

Recent data on trials using low-dose CYC, especially the Euro-Lupus Nephritis Trial, have raised the question of whether low-dose CYC be used instead of the high-dose regimen without sacrificing efficacy.

Doses are defined as follows:

- Low dose: 500 mg intravenously every 2 weeks for six doses, followed by maintenance with daily oral azathioprine or MMF.
- High dose: 750 g/m^2 intravenously every 4 weeks for 6 months, followed by maintenance with daily oral azathioprine or MMF.

The Euro-Lupus Nephritis Trial, a multicenter, randomized clinical trial, compared low and high doses (both added azathioprine) for remission induction in patients with severe disease. At 12 months, the probability of remission was approximately 50% in both groups, a statistically similar result [8].

Results continued to improve at a similar rate during a 60-month follow-up. Additionally, a 10-year follow-up of this trial found no differences in mortality, end-stage renal disease, or sustained doubling of serum creatinine [9]. These data clearly indicate that low-dose CYC provides sustainable results in European Caucasians.

One should note that these low-dose CYC studies were conducted in a European Caucasian population; patients from other races were not included. One therefore cannot conclude that low-dose CYC is effective in non-European Caucasians. However, clinical trials are in progress using populations from other races.

Adverse effects associated with low-dose CYC were not as high as those with high-dose CYC, but they were not zero. The most striking difference was with amenorrhea. The rate for the low-dose CYC group was similar to that in the general population in the European countries where this was studied.

Maintenance therapy

The Euro-Lupus Nephritis Trial also investigated maintenance therapy (after induction by CYC) with MMF or azathioprine during a 5-year study period [9]. The study population included both responders and nonresponders. Flares occurred in only about 20% of subjects, regardless of treatment – an impressive result. However, in a US clinical trial, MMF results were superior to azathioprine at 36 months for mortality, end-stage renal disease, serum creatinine, and major flares [10]. The study design was different in that all subjects enrolled in the maintenance trial had responded to the induction therapy. Nevertheless, it may be that MMF is a better choice than azathioprine for maintenance in responders.

The ACR guidelines for induction therapy for class III and class IV lupus nephritis recommend MMF at 2 to 3 g/day plus a steroid regimen, a guideline based on evidence from well-conducted randomized controlled trials. For Asians, however, 3 g/day was associated with relatively high rates of infection and mortality, so 2 g/day may be better as a top dose. For other racial populations, 3 g/day is recommended, if tolerated. The dosing for racial populations is based on opinion-level evidence rather than randomized trials.

For the steroid regimen, an intravenous pulse of glucocorticoids for 3 days is recommended, also an opinion-level guideline. A specific dose for daily prednisone was not recommended because data were not sufficient to make an evidence-based decision (Table 2); therefore, a range of 0.5 to 1.0 mg/kg/day was recommended.

The other option for induction is to use CYC plus steroids. With CYC, either low-dose or high-dose options can be used (these were discussed previously).

After 6 months, patients who show clinical improvement (for example, normalizing laboratory results, particularly complement, proteinuria and serum creatinine levels as well as an improved SELENA/SLEDAI score that might include lower scores for nephritis as well as mucocutaneous disease and arthritis) should be switched to maintenance therapy with either MMF or azathioprine. If patients are planning a pregnancy after they have improved, azathioprine is a better choice since MMF is teratogenic.

Patients who do not improve after 6 months on the initial regimen should be switched to the other drug regimen. For example, if the patient was on MMF therapy, then switch to CYC. If after another 6 months the response is still inadequate, then consider rituximab or calcineurin inhibitors along with glucocorticoids.

CLINICAL IMPLICATIONS

Teratogenicity

Several SLE therapies have been associated with fetal malformations. Data have linked these developmental abnormalities to MMF and to its active metabolite, myfortic acid, as well as to CYC and methotrexate. These drugs are contraindicated in any patient who is pregnant or is planning a pregnancy. In these patients, azathioprine is an option if there is a clinically defined need to suppress active lupus and glucocorticoids are

This presentation has not been subject to peer review. The statements and opinions expressed herein are those of the author, who bears full responsibility for the content of this presentation.

Table 2. Treatment of lupus nephritis classes III, IV, V

**Induction therapy**
MMF 2 to 3 g/day for 6 months (preferred in African Americans and Hispanics and for preserving fertility)
or
CYC intravenously for 6 months or until response
High dose: 500 to 1,000 mg/m^2/months×6 followed by daily azathioprine or MMF
Low dose: 500 mg every 2 weeks×6 followed by daily azathioprine or MMF
plus (either regimen)
Glucocorticoid intravenous pulse for 3 days
then
Prednisone 0.5 to 1 mg/kg/day tapered to lowest effective dose
Both regimens include hydroxychloroquine
**Maintenance therapy** (at 6 months)
MMF 1 to 2 g/day
or
Azathioprine 2 mg/kg/day with or without low-dose daily glucocorticoids
**Poor responders** (at 6 months)
Switch from MMF to CYC or *vice versa* for another 6 months
If response still inadequate, use rituximab or calcineurin inhibitor plus glucocorticoids

CYC, cyclophosphamide; MMF, mycophenolate mofetil.

not enough. Hydroxychloroquine can be used safely in SLE pregnancies and might prevent flares; if lupus is clinically active, addition of glucocorticoids and, if necessary, azathioprine is acceptable. The main problem associated with fetal loss in patients with SLE is active disease.

Predicting response

Treatment regimens for SLE take a relatively long time to work – 3 to 12 months is the average to see a clinical response. In the interim, how do practitioners gauge that a treatment is working? In a clinical trial comparing MMF with CYC, patients who showed at least a 25% reduction in proteinuria and improvement of C3 and/or C4 levels were three times more likely to have a clinical response to the treatment at 12 to 24 weeks. This indicates that one can measure the odds ratio of response by measuring these factors at 8 weeks without waiting 6 months to see a response.

Belimumab

As a treatment for SLE, belimumab has not been studied in patients with active renal disease or active central nervous system disease. A large phase 3 belimumab trial (BLISS-LN) [11] is being conducted. Among the 285 patients with renal disease who were included, there was no evidence that treatments worsened nephritis, and some parameters, such as proteinuria, were improved.

Data that are available suggest that belimumab can be considered in patients with active SLE that has persisted despite standard therapies. However, patients have to be able to wait 3 to 6 months for improvement because, by the nature of its mechanism of action (inhibition of maturation of B cells), it takes that long to determine whether belimumab is effective or not.

Investigational therapies in SLE

Drugs that target specific receptors and antigen presentations, which may have a role in SLE therapy, include the following:

- laquinimod, a quinoline;
- inhibitors of TLR7 and TLR9;
- inhibitors of interferon;
- anti-C5a, which interrupts complement fixation by the immune complexes;
- peptides that induce regulatory T cells of various types (for example, edratide, rigerimod);
- inhibitors of T-cell activation; and
- other approaches to suppress B-cell subsets.

Other therapies also are being investigated. A study on the use of *N*-acetyl cysteine, a nutritional supplement available at health-food stores, indicated that it might have the potential to improve lupus by dialing down T-cell hyperreactivity. There are products that interfere with T-cell and B-cell interaction (abatacept). There are new biologics (for example, epratuzumab), which have produced some exciting phase 2 data in terms of meaningful improvements in health-related quality of life measures over 4 years [12] and improvement in disease activity scores along with minimizing or eliminating concomitant corticosteroid use [13]. There are also targeted therapies such as anti-BLyS, TACI/Ig, anti-IL-6, and anti-TWEAK, a molecule of great interest as levels of TWEAK rise in the urine when lupus nephritis is flaring.

CONCLUSION

The search continues for a product that can inhibit both innate immunity and active adaptive immunity pathways in a manner that is effective and safe. Other pathways that govern damage, such as fibrosis, glomerular proliferation, and renal tubular cell injury, may also have to be inhibited to achieve better outcomes in patients with lupus nephritis.

ABBREVIATIONS

ACR, American College of Rheumatology; BLyS, B-lymphocyte stimulator; CYC, cyclophosphamide; MMF, mycophenolate mofetil; SELENA/SLEDAI, Safety of Estrogens in Lupus Erythematosus National Assessment/SLE Disease Activity Index; SLE, systemic lupus erythematosus.

REFERENCES

1. Hahn BH, McMahon MA, Wilkinson A, Wallace WD, Daikh DI, Fitzgerald JD, Karpouzas GA, Merrill JT, Wallace DJ, Yazdany J, Ramsey-Goldman R, Singh K, Khalighi M, Choi S, Gogia M, Kafaja S, Kamgar M, Lau C, Martin WJ, Parikh S, Peng J, Rastogi A, Chen W, Grossman JM: **American College of Rheumatology guidelines for screening, treatment, and management of lupus nephritis.**

This presentation has not been subject to peer review. The statements and opinions expressed herein are those of the author, who bears full responsibility for the content of this presentation.

Arthritis Care Res 2012, **64:**797-808.
2. Fessler BJ, Alarcon GS, McGwin G, Jr, Rosman J, Bastian HM, Friedman AW, Baethge BA, Vila L, Reveille JD; LUMINA Study Group: **Systemic lupus erythematosus in three ethnic groups: XVI. Association of hydroxychloroquine use with reduced risk of damage accrual.** *Arthritis Rheum* 2005, **52:**1473-1480.
3. Pons-Estel GJ, Alarcon GS, McGwin G, Jr, Danila MI, Zhang J, Bastian HM, Reveille JD, Vila LM; LUMINA Study Group: **Protective effect of hydroxychloroquine on renal damage in patients with lupus nephritis: LXV, data from a multiethnic US cohort.** *Arthritis Rheum* 2009, **61:**830-839.
4. Markowitz GS, D'Agati VD: **The ISN/RPS 2003 classification of lupus nephritis: an assessment at 3 years.** *Kidney Int* 2007, **71:**491-495.
5. Appel GB, Contreras G, Dooley MA, Ginzler EM, Isenberg D, Jayne D, Li L-S, Mysler E, Sanchez-Guerrer J, Solomons N, Wofsy D; Aspreva Lupus Management Study Group: **Mycophenolate mofetil *versus* cyclophosphamide for induction treatment of lupus nephritis.** *J Am Soc Nephrol* 2009, **20:**1103-1112.
6. Touma Z, Gladman DD, Urowitz MB, Beyene J, Uleryk EM, Shah PS: **Mycophenolate mofetil for induction treatment of lupus nephritis: a systematic review and metaanalysis.** *J Rheumatol* 2011, **38:**69-78.
7. Ginzler EM, Wofsy D, Isenberg D, Gordon C, Lisk L, Dooley MA; ALMS Group: **Nonrenal disease activity following mycophenolate mofetil or intravenous cyclophosphamide as induction treatment for lupus nephritis: findings in a multicenter, prospective, randomized, open-label, parallel-group clinical trial.** *Arthritis Rheum* 2010, **62:**211-221.
8. Houssiau FA, Vasconcelos C, D'Cruz D, Sebastiani GD, Garrido Ed Ede R, Danieli MG, Abramovicz D, Blockmans D, Mathieu A, Direskeneli H, Galeazzi M, Gül A, Levy Y, Petera P, Popovic R, Petrovic R, Sinico RA, Cattaneo R, Font J, Depresseux G, Cosyns JP, Cervera R: **Immunosuppressive therapy in lupus nephritis: the Euro-Lupus Nephritis Trial, a randomized trial of low-dose versus high-dose intravenous cyclophosphamide.** *Arthritis Rheum* 2002, **6:**2121-2131.
9. Houssiau FA, Vasconcelos C, D'Cruz D, Sebastiani GD, de Ramon Garrido E, Danieli MG, Abramovicz D, Blockmans D, Cauli A, Direskeneli H, Galeazzi M, Gül A, Levy Y, Petera P, Popovic R, Petrovic R, Sinico RA, Cattaneo R, Font J, Depresseux G, Cosyns JP, Cervera R: **The 10-year follow-up data of the Euro-Lupus Nephritis Trial comparing low-dose and high-dose intravenous cyclophosphamide.** *Ann Rheum Dis* 2010, **69:**61-64.
10. Dooley MA, Jayne D, Ginzler EM, Isenberg D, Olsen NJ, Wofsy D, Eitner F, Appel GB, Contreras G, Lisk L, Solomons N; ALMS Group: **Mycophenolate versus azathioprine as maintenance therapy for lupus nephritis.** *N Engl J Med.* 2011, **365:**1886-1895.
11. **Efficacy and Safety of Belimumab in Patients With Active Lupus Nephritis (BLISS-LN) [**http://www.clinicaltrials.gov/ct2/show/study/NCT01639339?term=belimumab&rank=23]
12. Strand V, Hobbs K, Wallace DJ, Kalunian K, Kilgallen B, Nikaı E, Wegener WA, Goldenberg DM: **Epratuzumab-treated systemic lupus erythematosus patients report improvements in health-related quality of life: final results from an open-label extension study (SL0006) [Abstract 2252].** *Arthritis Rheum* 2012, **64(Suppl):**S953.
13. Wallace DJ, Kalunian K, Petri MA, Strand V, Houssiau FA, Pike M, Kilgallen B, Bongardt S, Barry A, Kelley SL, Gordon C: **Efficacy and safety of epratuzumab in patients with moderate/severe active systemic lupus erythematosus: results from EMBLEM, a phase IIb, randomized, double-blind, placebo-controlled, multicentre study.** *Ann Rheum Dis* 2013. [Epub ahead of print]

This presentation has not been subject to peer review. The statements and opinions expressed herein are those of the author, who bears full responsibility for the content of this presentation.
